# Treatment of coronavirus disease 2019 in Shandong, China: a cost and affordability analysis

**DOI:** 10.1186/s40249-020-00689-0

**Published:** 2020-06-29

**Authors:** Xue-Zheng Li, Feng Jin, Jian-Guo Zhang, Yun-Feng Deng, Wei Shu, Jing-Min Qin, Xin Ma, Yu Pang

**Affiliations:** 1 School of Public Health, Cheeloo College of Medicine, Shandong University, Jinan, People’s Republic of China; 2 Provincial Key Laboratory for Respiratory Infectious Diseases in Shandong, Shandong Provincial Chest Hospital, Cheeloo College of Medicine, Shandong University, Jinan, People’s Republic of China; 3 Katharine Hsu International Research Center of Human Infectious Diseases, Shandon Provincial Chest Hospital, Cheeloo College of Medicine, Shandong University, No. 44 Wenhuaxi Road, Lixia District, 250012 Jinan, People’s Republic of China; 4 Department of Planning and Finance, Shandong Provincial Chest Hospital, Cheeloo College of Medicine, Shandong University, Jinan, People’s Republic of China; 5 National Clinical Laboratory on Tuberculosis, Beijing Key laboratory on Drug-resistant Tuberculosis Research, Beijing Chest Hospital, Capital Medical University, Beijing Tuberculosis and Thoracic Tumor Institute, No 9, Beiguan Street, Tongzhou District, Beijing, 101149 People’s Republic of China

**Keywords:** COVID-19, Cost, Treatment, Affordability, China

## Abstract

**Background:**

Coronavirus disease 2019 (COVID-19) is now a global public threat. Given the pandemic of COVID-19, the economic impact of COVID-19 is essential to add value to the policy-making process. We retrospectively conducted a cost and affordability analysis to determine the medical costs of COVID-19 patients in China, and also assess the factors affecting their costs.

**Methods:**

This analysis was retrospectively conducted in Shandong Provincial Chest Hospital between 24 January and 16 March 2020. The total direct medical expenditures were analyzed by cost factors. We also assessed affordability by comparing the simulated out-of-pocket expenditure of COVID-19 cases relative to the per capita disposable income. Differences between groups were tested by student *t* test and Mann-Whitney test when appropriate. A multiple logistic regression model was built to determine the risk factors associated with high cost.

**Results:**

A total of 70 COVID-19 patients were included in the analysis. The overall mean cost was USD 6827 per treated episode. The highest mean cost was observed in drug acquisition, accounting for 45.1% of the overall cost. Total mean cost was significantly higher in patients with pre-existing diseases compared to those without pre-existing diseases. Pre-existing diseases and the advanced disease severity were strongly associated with higher cost. Around USD 0.49 billion were expected for clinical manage of COVID-19 in China. Among rural households, the proportions of health insurance coverage should be increased to 70% for severe cases, and 80% for critically ill cases to avoid catastrophic health expenditure.

**Conclusions:**

Our data demonstrate that clinical management of COVID-19 patients incurs a great financial burden to national health insurance. The cost for drug acquisition is the major contributor to the medical cost, whereas the risk factors for higher cost are pre-existing diseases and severity of COVID-19. Improvement of insurance coverage will need to address the barriers of rural patients to avoid the occurrence of catastrophic health expenditure.

## Background

Coronavirus disease 2019 (COVID-19), caused by severe acute respiratory syndrome coronavirus 2 (SARS-CoV-2), is now a global public health threat [1, 2]. This virus has the potential to cause widespread pandemic with morbidity and mortality [3]. As of 30 April, 2020, a total of 3 090 445 human COVID-19 cases had been reported to the World Health Organization, including 84 373 cases from China and 3 006 072 cases from 212 countries and regions outside of China; of these, 217 769 were fatal [2]. In view of the striking increase in COVID-19 case numbers outside of China, WHO characterizes COVID-19 as a pandemic [2], highlighting the urgent need for international efforts to halt the rapid progression of its outbreak. In order to support the control of COVID-19, all patients were admitted to hospital in China regardless of disease severity. The Chinese government also declared the implementation of the financial support for COVID-19 since January 2020 (http://www.nhsa.gov.cn/art/2020/1/23/art_37_2284.html). The costs of medical care were primarily paid by health insurance, and the remaining costs were afforded by local government.

The spectrum of the COVID-19 severity is broad, ranging from asymptomatic to severe illness that requires mechanical ventilation [4]. In most infected people, COVID-19 is causing mild-to-moderate disease, whereas the mortality rate is markedly increased in the patients affected by severe disease [5]. Factors associated with severe presentation included elderly people and pre-existing comorbidities [6]. As a consequence, early initiation of proper therapy is essential for reducing the mortality associated with this disease. In in vitro screening studies, multiple agents were found to inhibit the SARS-CoV-2 production [7, 8]; however, there is limited evidence from randomized clinical trials to support any effective drug against COVID-19. In this context, antivirals and corticosteroids used in outbreaks of other coronaviruses are the major compassionate treatment options for COVID-19 cases [9]. In addition, prophylactic antibiotics are involved in treatment of severe cases to prevent secondary infection [6, 9]. The long-term administration of a dozen therapeutic drugs is associated with increased risk of adverse events, further complicating treatment regimens. For patients with critical illness, the application of unaffordable respiratory supporting devices (i.e. mechanical ventilation and extracorporeal membrane oxygenation) carries a high cost burden for patients and many funding systems [10]. Therefore, the costs of COVID-19 treatment vary drastically across individuals, depending on age, severity of disease, and comorbidity status. Given the pandemic of COVID-19, the economic impact of COVID-19 is essential to add value to the policy-making process. However, there is a paucity of studies aimed at quantifying the direct medical costs incurred by COVID-19 patients.

Shandong Province is located in eastern China, with a population of 100.7 million. As the second most populated province, it has fast economic development in the country. Despite not being close to Hubei Province, as of 3 May 2020, human-to-human transmission has driven its spread with a total of 788 confirmed cases, including 763 non-imported cases and 25 imported cases. To address this public health concern, we retrospectively conducted a cost and affordability analysis to determine the medical costs of COVID-19 patients in China, and also assess the factors affecting their costs.

## Methods

### Study design

This analysis was retrospectively conducted in Shandong Provincial Chest Hospital. This hospital is the only provincial-level tertiary hospital designated hospital for respiratory diseases in Shandong, including tuberculosis and other newly emerging respiratory illnesses, such as COVID-19. Between 24 January and 16 March 2020, 70 laboratory-confirmed patients consistent with national guidelines, including clinical symptoms, radiological abnormalities and positive molecular testing results for SARS-CoV-2 [11], were admitted in Shandong Provincial Chest Hospital. The patients were discharged from hospital when the criteria of cure have been met. “Cure” was defined as two consecutive negative molecular diagnostic results plus significant absorption of lesions and remission of clinical symptoms [5]. Demographic and clinical data were collected using patient medical records, while economic data was obtained from the financial management system. The study was approved by the Ethic Committee of Shandong Provincial Chest Hospital. Each patient provided informed consent prior to enrolment.

### Cost analysis

The costs of medical care were paid by health insurance and local government. Costs in this analysis were identified for drugs, radiological examinations, laboratory diagnostic tests, clinical measures, drug prescriptions, and hospitalization. Total costs were calculated from the following cost factors comprising the listed single positions: (i) laboratory diagnostic measures included microbiology, hematology, biochemistry, and blood gas analysis; (ii) radiographic measures included X-ray, computed tomography (CT) and B-mode ultrasonography; (iii) therapeutic measures included supplemental oxygen, hemopurification, salvage, and other measures used for treatment of COVID-19 and comorbidities; (iv) drug acquisition included all drugs indicated by the physician to be necessary for treatment of COVID-19 and comorbidities; (v) bed costs included costs of regular wards and intensive care units. Given that the medical cost in China was not separately accounted for staff expense, these expenditures were included in therapeutic measures. The costs of drugs were further divided according to their efficacy. Expenditures were calculated in US dollars (exchange rate: CNY 7.0 = USD 1.0).

### Affordability analysis

We assessed affordability by comparing the simulated out-of-pocket expenditure of COVID-19 cases relative to the per capita disposable income. The simulated out-of-pocket expenditure was calculated according to the reimbursement ratios by health insurance. In addition, we collected the rural and urban per capita disposable income of China from the National Bureau of Statistics (http://www.stats.gov.cn/), and family size according to the recent data from national population census (http://www.stats.gov.cn/ztjc/zdtjgz/zgrkpc/). The catastrophic expenditure was defined as the families who spend 50% or more of their disposable income [12]. High cost was defined as previously reported [13], arbitrarily as a cost equal to the 75% percentile.

### Statistical analysis

Demographic and treatment costs data were collected from electronic patient record system. Microsoft Excel 2013 (Microsoft Corp, Redmond, WA) was used for data entry. Statistical calculations were conducted using the statistical software SPSS 20.0 (IBM Corp, Armonk, NY). *P* value less than 0.05 was considered significant. The continuous variables were presented as means with standard derivations (SDs), while the categorical variables were expressed as percentages. A descriptive analysis was undertaken to compare the cost stratified to different patient groups. The comparison for normal variable was conducted using student *t* test. Mann-Whitney tests were performed for nonparametric data. Variables were introduced into a multiple logistic regression model if they were significantly associated with high cost at *P* < 0.10. The adjusted odds ratios (*OR*s) and 95% confidence interval (*CI*) were calculated according to this model.

## Results

### Demographic and clinical characteristics of the patients

A total of 70 COVID-19 patients were included in the analysis. All patients were treated in isolation. Demographic and clinical characteristics on hospital admission are shown in Table 1. Of 70 patients, 45 (64.3%) were male and 40 (57.1%) lived in urban region. The median age was 52 years (interquartile range: 37–65). Thirty-eight (54.3%) patients had pre-existing diseases. The most common pre-existing diseases was cardiovascular disease (21, 30.0%), followed by endocrine disease (12, 17.1%) and neurological disease (6, 8.6%). According to the national guidelines for disease severity grading, 55 (78.6%) were mild cases, 4 severe cases and 11 critically ill cases. Chinese medicine was noted in all the patients. 98.6% (69/70) and 61.4% (43/70) patients received antivirus and antibiotic treatment, respectively. In addition, immunomodulator (90.0%, 63/70) was another frequently used drug for COVID-19 treatment. Finally, 67 cases were cured, whereas the other 3 died of COVID-19.

**Table 1:** Demographic and clinical characteristics of patients in this study

Characteristics^**a**^	Patients (***n*** = 70)
**Sex (Male, %)**	45 (64.3)
**Age, years (Median, IQR)**	52 (37–65)
**Residence**	
Rural	30 (42.9)
Urban	40 (57.1)
**Pre-existing disease**	
Any pre-existing disease	38 (54.3)
Cardiovascular disease	21 (30.0)
Endocrine disease	12 (17.1)
Neurological disease	6 (8.6)
Rental disease	4 (5.7)
Respiratory disease	3 (4.3)
Liver disease	1 (1.4)
Malignancy	1 (1.4)
**Disease Severity**	
Mild	55 (78.6)
Severe	4 (5.7)
Critically ill	11 (15.7)
**Drug acquisition**	
Antivirus	69 (98.6)
Antibiotic	43 (61.4)
Antifungal	8 (11.4)
Immunomodulator	63 (90.0)
Pre-existing diseases	38 (54.3)
Chinese medicine	70 (100.0)
**Clinical outcomes**	
Cure	67 (95.7)
Died	3 (4.3)
**Duration of hospitalization, days (Median, IQR)**	16 (10–23)

^a^*IQR* interquartile range

### Cost analysis

**Fig. 1:**
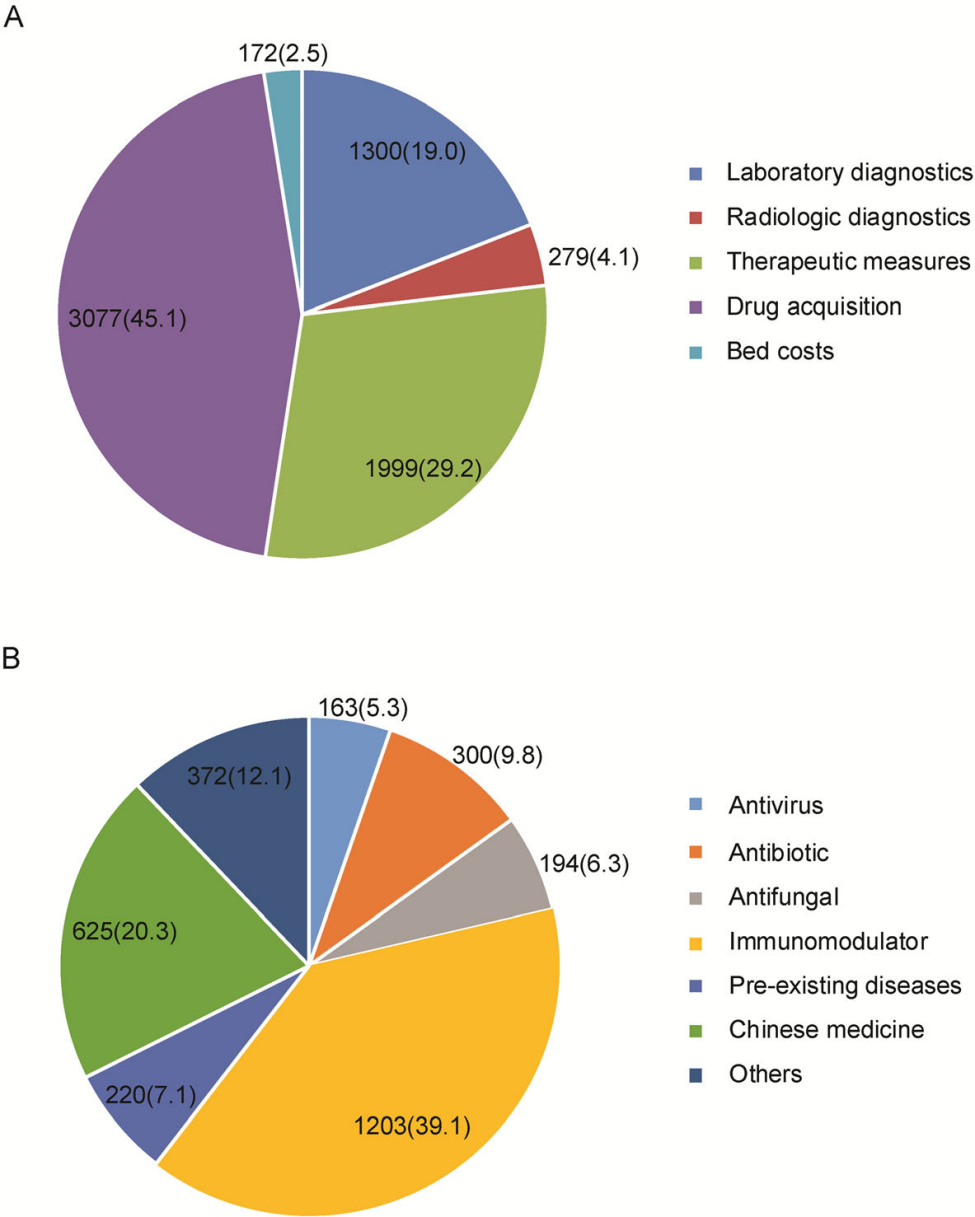
Composition of medical cost per treated episode of COVID-19. **a** Composition of medical cost per treated episode of COVID-19, USD (%); **b**. Cost of drug acquisition stratified by drug efficacy, USD (%)

**Fig. 2:**
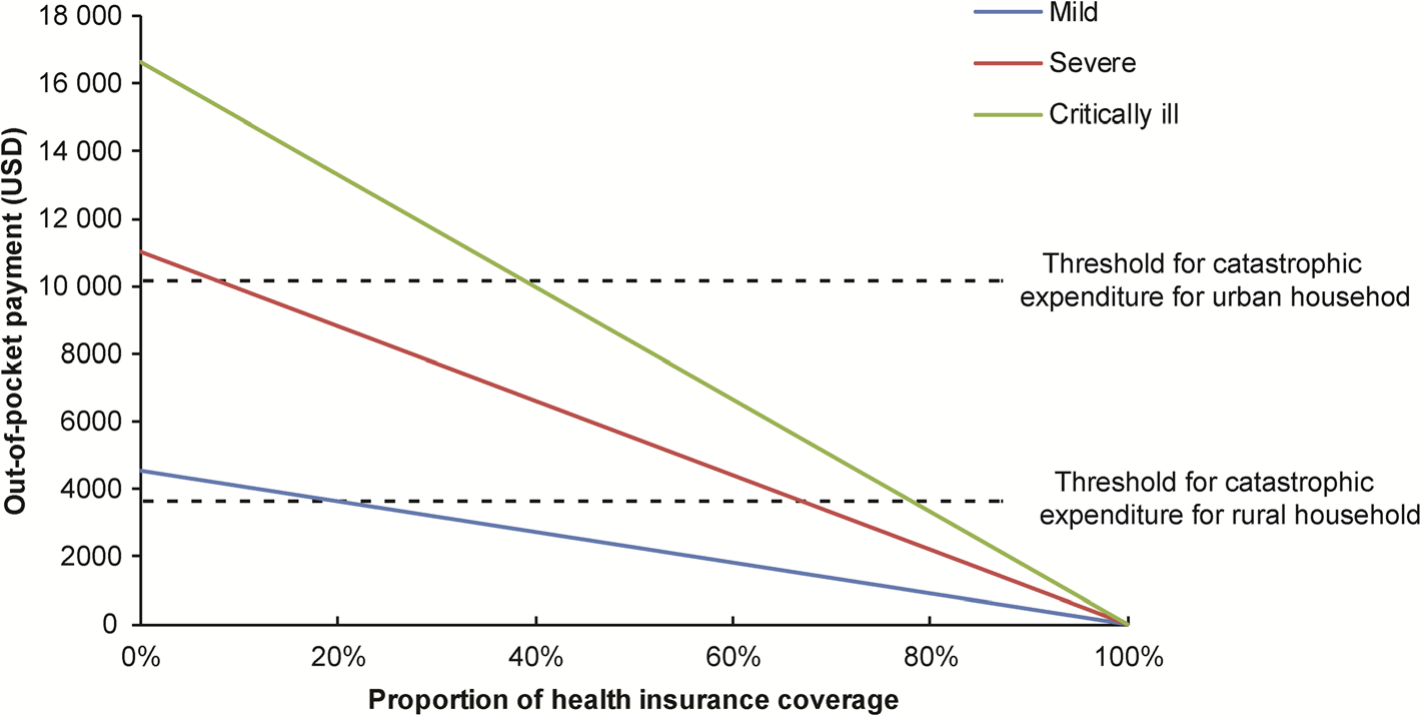
Modeling of the catastrophic health expenditures due to COVID-19 disease stratified by disease severity. The threshold for catastrophic expenditure for household is estimated by 50% of the disposable income of household. The disposable income is calculated as follow: per capita disposable income (USD 5606 for urban household and USD 2056 for rural household) multiplies the average family size in 2019 (3.35 members per family)

The mean costs for clinical management of COVID-19 patients are summarized in Fig. 1a. The overall mean cost was USD 6827 per treated episode of COVID-19. The highest mean cost was observed in drug acquisition (USD 3077), accounting for 45.1% of the overall cost. Total mean costs for laboratory diagnostics and therapeutic measures were USD 1300 (19.0%) and USD 1999 (29.2%), respectively. Given that the largest factor driving total treatment costs was drug acquisition, we further analyzed the cost of drug acquisition according to drug efficacy. As shown in Fig. 2b, mean values were USD 1203, USD 625, and USD 300 for immunomodulator, Chinese medicine, antibiotic, respectively.

The total mean cost for clinical management of COVID-19 according to various demographic and clinical characteristics is summarized in Table 2. Total mean cost was significantly higher in patients with pre-existing diseases (USD 9525) compared to those without pre-existing diseases (USD 3619, *P* < 0.001). The increasing trend in mean total cost was observed with advanced age, ranging from USD 2752 for 0–34 years group to USD 11668 for ≥ 70 years group (*P* = 0.002), which majorly contributed to the greater odds for pre-existing diseases in elderly patients. We also found that the mean cost was associated with the disease severity, the value of critically ill cases (USD 16 652) was significantly higher than that of mild cases. In contrast, the sex and residence had no significant effect on the total cost. Notably, the statistically higher expenses in patients with pre-existing diseases were identified in all individual cost factors investigated in this study (*P* < 0.01) (Table 3).

**Table 2:** Comparison of cost for clinical management of COVID-19 patients stratified by various demographic and clinical characteristics

Characteristics	Cost per case^**a**^ (USD, Mean ± SD)	***P*** value
**Sex**		**0.966**
Male	6637 ± 7040	
Female	7165 ± 7847	
**Age group (years)**		**0.002**
0–34	2752 ± 2016	
35–69	6850 ± 7339	
70–	11 668 ± 8584	
**Residence**		**0.280**
Rural	8052 ± 8458	
Urban	5905 ± 6221	
**Pre-existing disease**		**< 0.001**
No	3619 ± 3243	
Yes	9525 ± 8592	
**Severity**		**< 0.001**
Mild	4552 ± 4831	
Severe	11 058 ± 9329	
Critically ill	16 652 ± 8506	

^a^*USD* US dollar; *SD* standard deviation

**Table 3:** Comparative cost analysis for clinical management of COVID-19 case with and without underlying diseases

Classification	Cost per case (USD, Mean ± SD)		***P*** value
	Without underlying diseases	With underlying diseases	
**Laboratory diagnostics**	948 ± 536	1595 ± 856	0.001
**Radiologic diagnostics**	216 ± 124	332 ± 172	0.001
**Therapeutic measures**	951 ± 920	2882 ± 2940	0.001
**Drug acquisition**	1375 ± 1832	4511 ± 4937	< 0.001
**Bed costs**	129 ± 53	208 ± 127	0.003
**Total**	3619 ± 3243	9525 ± 8592	0.001

A regression model was modeled to identify variables independently associated with high cost (USD 6877). The results of the regression model are presented in Table 4. Pre-existing diseases was strongly associated with higher cost (*OR* = 33.70, 95% *CI*: 1.66–682.99). In addition, the advanced disease severity was also associated with higher cost (*OR* = 144.90, 95% *CI*: 3.79–5537.64 for severe cases; *OR* = 64.12, 95% *CI*: 5.89–697.73 for critically ill cases).

**Table 4:** Variables in the model for high direct medical cost (> USD 6877 per case)

Variables	Estimated adjusted ***OR***^**a**^	95% ***CI***
**Pre-existing disease**	33.70	1.66–682.99
**Severity**		
**Severe**	144.90	3.79–5537.64
**Critically ill**	64.12	5.89–697.73
**Age**	0.98	0.92–1.05

^a^*OR* odds ratio, *CI* confidence interval

### Estimated financial burden

By 21 March 2020, 81 416 laboratory-confirmed COVID-19 cases were reported in China (http://www.nhc.gov.cn/xcs/yqtb/list_gzbd.shtml). The number of cases with varying severity was estimated by the recent report from Chinese Center for Disease Control and Prevention, demonstrating that the proportion of mild, severe and critically ill cases were 81, 14 and 5%, respectively. On the basis of these estimates, the total burdens were USD 0.30 billion (61%) for mild cases, USD 0.13 billion (26%) for severe case, and USD 0.07 billion (13%) for critically ill cases, respectively. In total, around USD 0.49 billion were expected for clinical manage of COVID-19, and financed a significant proportion of the proposed national health care plan (about 0.2% of the China health care expenditure in 2019) (Table 5).

**Table 5:** Estimated financial burden to the national health insurance for COVID-19 patients in China

Characteristics	Estimated number of cases^**a**^	Cost per case (USD)	Total cost of COVID-19 cases (USD)
**Mild**	65 947 (81%)	4552	300 190 744 (61%)
**Severe**	11 398 (14%)	11 058	126 039 084 (26%)
**Critically ill**	4071 (5%)	16 652	67 790 292 (13%)
**Total**	81 416 (100%)	**–**	494 202 120 (100%)

^a^The number of COVID-19 cases were referenced according to the national surveillance report by the National Health Committee by 21 March 2020. The proportion of cases with varying severity was confirmed according to the Chinese Center for Disease Control and Prevention Report

– Not applicable

We modelled the potential catastrophic health expenditure due to COVID-19 disease stratified by disease severity. As shown in Fig. 2, when the proportion of health insurance coverage achieved 10% for severe case and 40% for critically ill case, respectively, the urban households could escape from catastrophic health expenditure at estimated threshold value. Among rural households, the proportions should be increased to 70% for severe cases, and 80% for critically ill cases to avoid catastrophic health expenditure (Fig. 2).

## Discussion

An accurate understanding of cost structures is essential for clinical management of COVID-19 patients. Our data demonstrate that the overall mean cost was USD 6827 per treated episode of COVID-19, which was significantly higher than those of influenza (about USD 25), community-acquired pneumonia (about USD 650) and severe acute respiratory syndrome (about USD 2700) in China [14–16]. The diversity in medical expenditure majorly contributed to the aggressive treatment measure for COVID-19 cases in China. On one hand, the lack of specific drugs against this virus necessitates the use of drugs with limited evidence for COVID-19, and multiple additional drugs for prophylactic pathogen infections [17], thereby increasing the total medical cost. On the other hand, in view of high potential for acute progression [18], the more frequent monitoring of laboratory tests and radiography examinations were conducted to evaluate the clinical response for patients, which may be another explanation for the high expenditure in COVID-19 patients.

Of note, drug acquisition accounts approximately half of total direct cost in our cohort, of which immunomodulator, Chinese medicine and antibiotic occupy the top three rank positions by mean cost per case. In our opinion, this cost rank is associated with the diversity of unit price of each drug. Intravenous immune globulin therapy is the predominant immunomodulator used in the management of patients with COVID-19 [19]. Despite exhibiting satisfactory clinical benefit for clinical remission, its high cost is of concern to patients and health insurance systems in the future [20]. As the second contributor, the role of Chinese medicine in treatment of COVID-19 remains controversial [21, 22]. Primary clinical experience indicated that the early intervention of Chinese medicine could impede further development of COVID-19 severity, and shorten its clinical course [22]. Further study is urgently required to determine cost-effectiveness of Chinese medicine as adjuvant therapy in the controlled randomized trials.

As expected, the patients with pre-existing diseases are strongly associated with higher cost than those without pre-existing diseases. The reasons for this observation are multifactorial. First, there is no doubt that the use of drugs for pre-existing diseases will increase the medical expense. Second, the concurrent diseases at admission imply more severity and complexity. Several studies documented that the comorbidities are recognized as independent factors in increased disease severity [23, 24], thus resulting in prolonged length of hospital study and enhanced therapy measure that dramatically increase the costs of managing COVID-19 patients. In view of the extreme high cost of medical cost, early interventions are of great clinical importance for reducing the financial burden in this population.

Although the costs of medical care were paid by health insurance and local government in China, we constructed an estimated model taking the household income and health insurance scheme into consideration to assess the affordability for clinical management of COVID-19. For urban households, the proportion of insurance payment is about 70% of total in-hospital payment [25]. In this context, the urban households are less likely to incur catastrophic health expenditure at present cut-off value. By contrast, the proportion of health insurance coverage should be achieved at 80% for critically ill cases who could escape from catastrophic health expenditure for the tentative threshold. However, under the current health insurance scheme for rural household, patients have to afford approximate half of total medical cost [26]. Thus, there is an obvious gap between social requirement and current situation in health insurance. Improvement of insurance coverage will need to address the barriers of rural patients to avoid the occurrence of catastrophic health expenditure.

There were several obvious limitations in this study. First, despite enrolment of all COVID-19 cases in our hospital, the small sample size from the single center may weaken the significance of our conclusion. Second, the present study excluded partial direct costs to patients and indirect costs, such as lost working days. Therefore, the total cost of COVID-19 patients is underestimated. Third, the insurance coverage and health benefits were significantly different across different regions in China. Hence, this study was more likely to be a theoretical cost analysis in view of that fact that Chinese government affords medical expenditures for COVID-19 patients. Fourth, despite exhibiting promising efficacy for treatment of patients infected with COVID-19, the traditional Chinese medicines are composed of several classical herbal materials for exogenous fever. Thus, it is difficult to elucidate the systemic pharmacological mechanisms of action of Chinese medicine against SARS-CoV-2. Finally, drug prices can be expected to change substantially across regions. This diversity may lead to potential bias in estimation of national financial burden. Nevertheless, our study firstly provides important insights in direct medical cost of COVID-19 patients in China.

## Conclusions

Our data demonstrate that clinical management of COVID-19 patients incurs a great financial burden to national health insurance. The cost for drug acquisition is the major contributor to the direct medical cost, whereas the risk factors for higher cost are pre-existing diseases and severity of COVID-19. Despite government financial support to clinical management of COVID-19 patients, an estimated model indicates that improvement of insurance coverage will need to address the barriers of rural patients to avoid the occurrence of catastrophic health expenditure.

## Data Availability

The datasets generated and analyzed from the current study are not publicly available at this time as further analyses are ongoing, but are available from the corresponding author on reasonable request.
